# Tumor-Targeted Human T Cells Expressing CD28-Based Chimeric Antigen Receptors Circumvent CTLA-4 Inhibition

**DOI:** 10.1371/journal.pone.0130518

**Published:** 2015-06-25

**Authors:** Maud Condomines, Jon Arnason, Reuben Benjamin, Gertrude Gunset, Jason Plotkin, Michel Sadelain

**Affiliations:** Center for Cell Engineering and Molecular Pharmacology and Chemistry Program, Memorial Sloan-Kettering Cancer Center, New York, NY, 10065, United States of America; Saint Louis University School of Medicine, UNITED STATES

## Abstract

Adoptive T cell therapy represents a promising treatment for cancer. Human T cells engineered to express a chimeric antigen receptor (CAR) recognize and kill tumor cells in a MHC-unrestricted manner and persist *in vivo* when the CAR includes a CD28 costimulatory domain. However, the intensity of the CAR-mediated CD28 activation signal and its regulation by the CTLA-4 checkpoint are unknown. We investigated whether T cells expressing an anti-CD19, CD3 zeta and CD28-based CAR (19-28z) displayed the same proliferation and anti-tumor abilities than T cells expressing a CD3 zeta-based CAR (19z1) costimulated through the CD80/CD28, ligand/receptor pathway. Repeated *in vitro* antigen-specific stimulations indicated that 19-28z^+^ T cells secreted higher levels of Th1 cytokines and showed enhanced proliferation compared to those of 19z1^+^ or 19z1-CD80^+^ T cells. In an aggressive pre-B cell leukemia model, mice treated with 19-28z^+^ T cells had 10-fold reduced tumor progression compared to those treated with 19z1^+^ or 19z1-CD80^+^ T cells. shRNA-mediated CTLA-4 down-regulation in 19z1-CD80^+^ T cells significantly increased their *in vivo* expansion and anti-tumor properties, but had no effect in 19-28z^+^ T cells. Our results establish that CTLA-4 down-regulation may benefit human adoptive T cell therapy and demonstrate that CAR design can elude negative checkpoints to better sustain T cell function.

## Introduction

Adoptive T cell therapy using genetically modified autologous T cells is beginning to show promising results in patients with melanoma and indolent B cell malignancies [1–5]. In particular, human T cells engineered to express a chimeric antigen receptor (CAR) that is specific for CD19 [6], a B cell surface antigen, are emerging as a paradigm and a broadly investigated test case for CAR technology [7]. CARs incorporate an scFv derived from an antibody or, alternatively, a Fab selected from recombinant libraries, fused to the CD3ζ chain, and thus provide an MHC unrestricted first signal of activation [8]. First generation CARs which only provide a CD3ζ activation signal [9], direct limited T cell proliferation in the absence of costimulation and are prone to T cell anergy [10,11] resulting in reduced T cell persistence upon transfer to cancer patients [12]. Multiple studies indicate that costimulatory signals are needed for CAR-targeted T cells to avoid anergy, to be fully activated and sustain their expansion [6,13–16]. Costimulation may be provided independently of the CAR, for example through the CD28 receptor and CD80/CD86 interactions [6,15,17] or through the CAR itself, as exemplified by second-generation CARs encompassing the CD28 cytoplasmic domain in addition to a T cell activation domain [13]. We and others showed that a CAR embedding the CD28 signaling domain triggers less apoptosis, higher AKT/PI3K activation and IL-2 secretion than CD3 zeta-based CARs, while displaying similar cytotoxicity [13,17,18]. Furthermore, CD19-targeted T cells harboring a second generation CAR (19-28z) promote higher tumor rejection rate than T cells expressing a first generation CAR (19z1) [14,19]. Thus, CD28-based CARs can provide to T cells more than a mere activation signal without requiring the CD28 ligands CD80/CD86. However, the magnitude of the CAR-mediated CD28 signal obtained in T cells has not been extensively compared to that provided by the interaction of CD80/CD86 with endogenous CD28 receptors. Notably, CD80 and CD86 also bind CTLA-4, a CD28 homolog, which strongly inhibits T-cell activation [20]. Whereas CTLA-4 is well known to dampen effector T cell function, regulate homeostatic lymphoproliferation and induce tolerance, its effect on adoptively transferred tumor-targeted human T cells, including T cells that are costimulated through a second generation CAR, is presently unknown.

Phenotypically, CTLA-4 engagement results in cell cycle arrest and inhibition of T-cell proliferation [20]. In primary T cells, CTLA-4 is recruited at the immunological synapse soon after TCR engagement [21] but how it dampens T-cell response is still not fully elucidated. Several mechanisms of action of CTLA-4 have been described [22] including competition of the extracellular domain with CD28 for ligand binding [23], blockade of lipid raft surface expression [24], decrease of TCR molecules’ accumulation in lipid rafts [25], reversal of the TCR stop signal [26] and ligand trans-endocytosis [27]. Yokosuda *et al* emphasized the role of physical stabilization of CTLA-4 by CD80 at the immunological synapse [28]. The existence of intracellular signaling pathways induced by CTLA-4 is under debate in the literature [29]. Notably, the expression of a tailless CTLA-4 molecule prevents lethal lymphoproliferation in CTLA-4-/- mice [30] and mice with CTLA-4 mutated in its intracellular domain don’t develop autoimmune diseases [31].

We previously reported that T cells expressing a CD3zeta-based CAR along with CD80 undergo auto-costimulation achieved through the recruitment of CD80 and CD28 molecules at the immunological synapse [15]. The overall dependence of adoptively transferred T cells on costimulation, together with the potential to bypass physiological checkpoint circuits using second generation CARs, led us to hypothesize that CD19-targeted T cells that depend on their endogenous CD28 receptor to sustain their activity are constrained by CTLA-4 inhibition but that 19-28z expressing T cells are less so. To investigate this question, we took advantage of the auto-costimulation system to compare CD28-mediated and CD28-based CAR-mediated tumor rejection, and examined whether down-regulation of CTLA-4 in human peripheral blood T cells would improve their anti-tumor capacities in the context of adoptive T cell therapy. We show here that 19-28z^+^ T cells secrete more IL-2, survive longer and exhibit superior *in vivo* anti-tumor activity than do 19z1-CD80^+^ T cells. Down-regulation of CTLA-4 significantly increased the *in vivo* anti-tumor effect of 19z1-CD80^+^ T cells but not that of 19-28z^+^ T cells. These results demonstrate that the potency of adoptively transferred, CD19-targeted T cells dependent of CD28/CD80-costimulation, is increased by CTLA-4 blockade and establish for the first time that 19-28z^+^ T cells, which prolong survival of animals bearing aggressive leukemia, are not sensitive to CTLA-4 inhibition.

## Material and Methods

### Cell lines

Wild type NALM/6 tumor cell line and NALM/6 expressing firefly luciferase-GFP, described previously [19], were cultured in RPMI 1640 (Life Technologies) supplemented with 10% heat-inactivated FCS, nonessential amino acids, HEPES buffer, pyruvate, and BME (Life Technologies). 293T cell line, H29 and retroviral packaging cell lines were cultured in DMEM (Life Technologies) supplemented with 10% FCS. NIH-3T3 artificial antigen-presenting cells (aAPCs), described previously [6], were cultured in DMEM supplemented with 10% heat-inactivated donor calf serum. All media were supplemented with 2 mmol/L l-glutamine (Life Technologies), 100 units/mL penicillin, and 100 μg/mL streptomycin (Life Technologies).

### Retroviral vector constructs, shRNAs and retroviral production

Plasmids encoding the SFG oncoretroviral vector were prepared using standard molecular biology techniques. Syntheses of SFG-19z1-ΔLNGFR, SFG-19-28z, SFG-CD80 and SFG-DsRED have been previously described [13,32]. The SFG-19z1-ΔLNGFR plasmid that includes a P2A bicistronic element was used as a template to obtain SFG-19z1-CD80 and 19-28z-ΔLNGFR constructs.

We PCR amplified the cDNA encoding human CTLA-4 3’ UTR sequence in with cDNA from PHA-activated human PBMC. We constructed a bicistronic CTLA-4-GFP sequence using the IRES peptide sequence as previously described [33] and cloned it into the P-CAG plasmid (Clontech).

A set of small hairpin RNAs (shRNA) targeting CTLA-4 (GeneBank accession numbers NM_001037631 and NM_005214) designed by Open Biosystems (Clone Id V2HS-43695, V2HS-43692, V2HS-243320, termed here as shRNA#1, shRNA#2 and shRNA#3, respectively) were synthesized, annealed and cloned in a U6 transcription cassette inserted into the 3’ long term repeat (LTR) of SFG-CD80 or SFG-DsRED, as described [15]. We used a control shRNA targeting the kanamycin gene, kindly provided by Dr. L. Lisowski, with the following sequence:

5’TTGACAGTGAGCGCGAGAAATCACCATGAGTGACGACGTGAAGCCACAGATGGTCGTCACTCATGGTGATTTCTCATGCCTACTGCCTCGGA-3’ (sense),

5’TCCGAGGCAGTAGGCATGAGAAATCACCATGAGTGACGACCATCTGTGGCTTCACGTCGTCACTCATGGTGATTTCTCGCGCTCACTGTCAA-3’ (antisense).

VSV-G pseudotyped retroviral supernatants derived from transduced gpg29 fibroblasts (H29) were used to construct stable retroviral producing cell lines using polybrene (Sigma), as previously described [10,34].

### Human T cell cultures and retroviral transduction

Blood samples were obtained from healthy volunteers who provided written informed consent under research blood sample protocols reviewed and approved by Memorial Sloan-Kettering Cancer Center’s Institutional Review Board. PBMC were isolated by low-density centrifugation on Lymphoprep (Accurate Chemical and Scientific Corporation, Westbury, NY), activated with PHA for 48h and transduced on two consecutive days by centrifugation on retronectin-coated (Takara), oncoretroviral vector-bound plates. Seven days after PHA stimulation, transduced T cells were stained for transduction rate measurements and either injected to tumor-bearing mice or cocultured with irradiated confluent CD19^+^ or CD19^+^CD80^+^ NIH 3T3 aAPCs, at 10^6^ cells/ml in 24-well tissue culture plates in RPMI medium supplemented with 10% FCS, l-glutamine, streptomycin, and penicillin, with no added cytokines. Identical stimulations in fresh medium were performed weekly. Supernatants were harvested 24h after T-cell stimulation for cytokine detection. T-cell cultures were supplemented with fresh medium to maintain a concentration of 1.5–2 x 10^6^ cells/ml.

### Flow cytometry

Flow cytometry was performed on a BD-LSRII cytometer and data analyzed with FlowJo software (Treestar). The following mAbs were used for phenotypic analysis: phycoerythrin (PE)-labeled anti human LNGFR, PE-Cy5.5 anti-human CD3, PE- or allophycocyanin (APC)-labelled anti-human CTLA-4 (BD Biosciences), APC-conjugated goat anti mouse IgG used for CAR detection, pacific blue-labeled anti-human CD8 and PE-Cy7-labeled anti-human CD4 (Caltag/Invitrogen). Intracellular staining of CTLA-4 was performed using the Cytofix/Cytoperm kit (BD Pharmingen), according to the manufacturer’s instructions.

### Cytokine detection assays

Cytokine concentrations were measured in T-cell cultures by using a custom multiplex system HCYTMAG-60K (Millipore), according to the manufacturer’s instructions. Luminescence was assessed using the Luminex IS100 device and analyzed for cytokine concentration using IS 2.2 software (Luminex Corp.).

### Assessment of shRNA efficacy

ShRNAs containing Ds-Red SFG plasmids were costransfected with CTLA-4-GFP containing plasmids using the CaCl_2_ transfection method at a 10:1 ratio in 293 T cells. 48h later, cells were analyzed by flow cytometry and GFP mean fluorescence intensities (MFIs) were determined within shRNA reporter gene gates.

### Quantitative Real-Time PCR

RNA was extracted with the RNeasy Micro Kit (QIAGEN) and reverse transcribed into cDNA by oligo-dT primer (Invitrogen) and Superscript First Strand Synthesis System (Invitrogen). Real-time PCR was completed with TaqMan gene expression assays Hs00175480_m1 CTLA-4, Hs00984230_m1 B2M and the TaqMan Universal Master Mix from Applied Biosystems using the ABI Prism 7500 sequence detection system. Quantitative PCR analysis was done using ABI Prism 7500 SDS software. *C*_t_ values were collected during the log phase of the cycle. CTLA-4 levels were normalized to B2M for each sample (Δ*C*_t_ = *C*_t_ gene of interest − *C*_t_ B2M) and relatively compared using the following formula 100/^2ΔΔ*C*t^, where 2ΔΔ*C*_t_ = Δ*C*_t_ unknown − Δ*C*_t_-reference.

### Mouse systemic tumor model

We used 8- to 12-week old NOD.Cg-Prkdc^scid^Il2rg^tm1Wjl^/SzJ (NSG) mice purchased from the Jackson Laboratories (Bar Harbor, ME) or house inbreed, under a protocol approved by the Memorial Sloan-Kettering Cancer Center’s Institutional Animal Care and Use Committee (protocol # 04-10-024). Mice were inoculated with 0.5x10^6^ FF-luc-GFP NALM/6 cells by tail vein injection followed by 2x10^5^ CAR^+^ T cells four days later. Mice were euthanized when they developed hind-limb paralysis or were otherwise moribund.

For bone marrow T cell counts, mice were sacrificed and two femurs per mouse homogenized using pestle and mortar in 5 ml medium, and then filtered using a 40-μm cell strainer. Red blood cells were lysed using ACK lysing buffer (LONZA) and the cells were re-suspended in PBS. Cells were counted, stained and T cell percentages determined by flow cytometry. When indicated, circulating CAR T cells counts were measured using BD Trucount beads on stained 50μl blood samples.

### *In vivo* quantitative bioluminescence of NALM/6 tumors

Mice were infused by i.p. injection with 150 mg/kg of d-luciferin (Xenogen) suspended in 200 μL of PBS. Ten minutes later, mice were imaged while under 3% isoflurane anesthesia. Bioluminescence imaging was done using Xenogen IVIS Imaging System (Xenogen) with Living Image software (Xenogen) for acquisition of imaging data sets. Image acquisition was done on a 20-cm field of view at medium binning level for 0.2- to 2-min exposure time. Both dorsal and ventral views were obtained on all animals. Tumor burden, as determined by IVIS imaging, was assessed as described previously [35].

### Statistical analyses

Data were analyzed using Prism 5.0 (GraphPad Software, Inc.). Statistical comparisons between two groups were determined by a Student’s *t* test. All *p* values are two-tailed. The Log-rank test was used to compare survival curves obtained with Kaplan-Meier method.

## Results

### T cells expressing the 19-28z CAR display higher proliferation and produce higher levels of type 1 cytokines in response to CD19 than CD80-costimulated T cells expressing the 19z1 CAR

**Fig 1:**
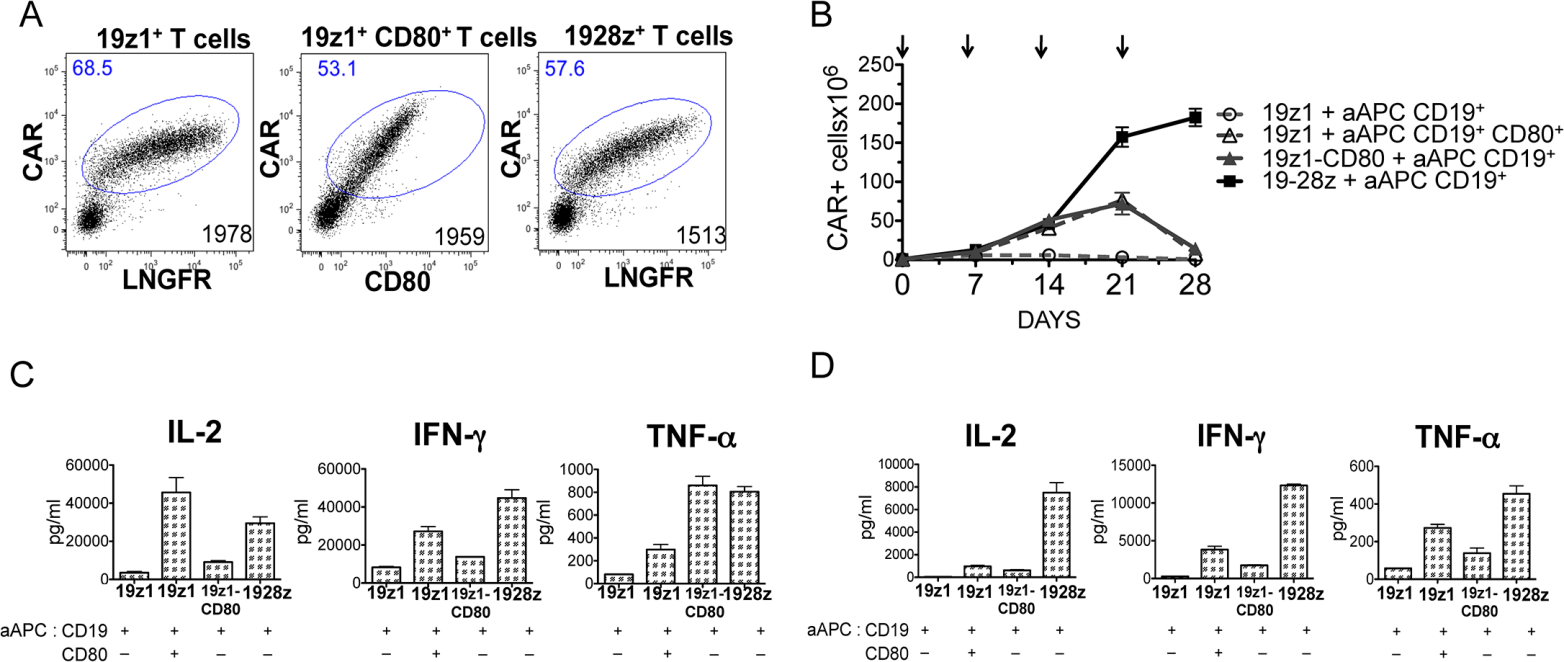
Different CD28 costimulation for CD3z/CD28-based CAR or CD3z-based CAR + CD80 transduced T cells. (A) Plots represent CAR transduction efficiencies in primary T cells, measured by flow cytometry 4 days after retroviral transduction. Numbers in upper left quadrants indicate percentages of transduced cells; numbers in lower right quadrants indicate CAR MFIs. Since ΔLNGFR is used as a technical control and has no functional impact on T cells, it is omitted in the termed T-cell groups for clarity. (B) *In vitro* T cell growth of 19z1^**+**^, 19z1-CD80^**+**^ and 19-28z^**+**^ T cells weekly cocultured with artificial antigen presenting cells (aAPC) expressing CD19 or CD19 and CD80, as indicated, without exogenous cytokines. CAR expression was weekly determined by flow cytometry. Arrows indicate stimulation time points. (C, D) Cytokine concentrations measured in supernatants of cocultures depicted in B, at day 8 (C) and day 22 (D). These data are representative of one out of three different experiments producing similar patterns.

Human primary T cells were transduced with bicistronic retroviral vectors encoding the 19z1 CAR and ΔLNGFR, 19z1 and CD80 or the 19-28z CAR and ΔLNGFR with a similar gene transfer level (Fig 1A). We used ΔLNGFR, a mutant transmembrane protein inactive in T cells [33], as a technical control molecule to ensure comparable CAR expression levels from different bicistronic vectors. Upon repeated *in vitro* antigen specific stimulations on artificial antigen presenting cells (aAPC) expressing CD19, without exogenous cytokines, 19-28z^+^ T cells proliferated and survived longer than non-costimulated 19z1^+^ T cells (Fig 1B). 19z1^+^ T cells died soon after the first stimulation, as previously observed [15,19], confirming that costimulation is necessary for persistence of cultured CAR-targeted T cells. We then compared the antigen-triggered growth of 19-28z^+^ T cells to that of 19z1^+^ T cells receiving CD80 costimulation in two different ways, with CD80 either expressed by the T cells or by the aAPCs. 19-28z^+^ T cells showed similar proliferation rates to that of CD80-costimulated 19z1^+^ T cells during 14 days but displayed greater survival after the third stimulation, whether CD80 was provided by the T cells or by the aAPCs (Fig 1B). IL-2, IFNγ and TNFα levels measured in the culture supernatants showed a sustained cytokine secretion for 19-28z^+^ T cells and exhaustion for CD80-costimulated 19z1^+^ T cells. Non-costimulated 19z1^+^ T cells secreted the lowest amounts of cytokines at all time points (Fig 1C and 1D).

### 19-28z^+^ T cells exhibit more potent anti-tumor capacity than 19z1-CD80^+^ T cells in a xenograft model of aggressive acute lymphoblastic leukemia

**Fig 2:**
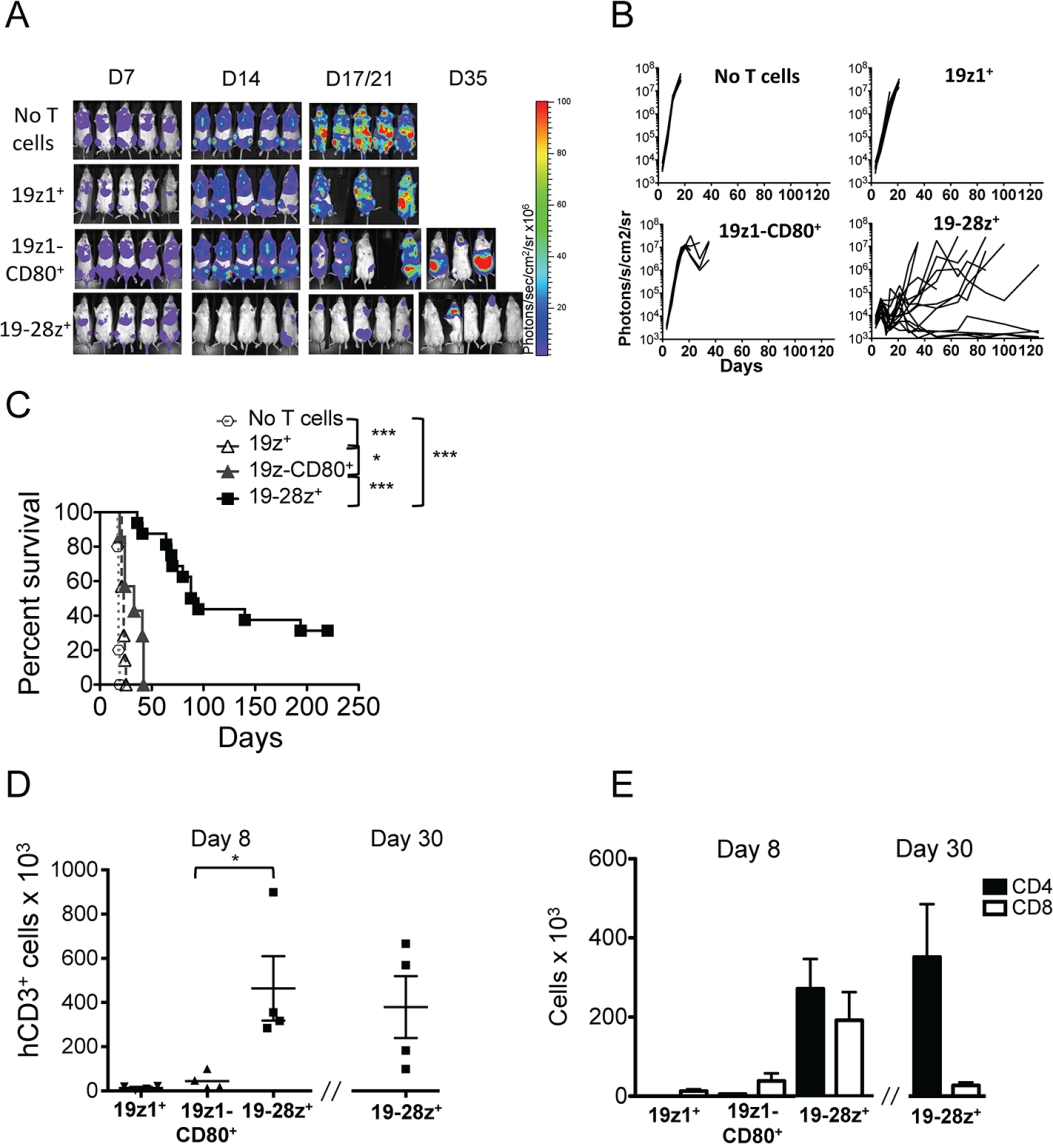
*In vivo* anti-leukemia abilities of 19-28z^+^, 19z1^+^ and 19z1-CD80^+^ T cells. (A) Representative ventral bioluminescence images of GFP-FFLuc NALM/6 bearing NSG mice non treated or injected with 2x10^**5**^ CAR^**+**^ 19z1^**+**^, 19z1-CD80^**+**^ or 19-28z^**+**^ human T cells. Signal intensities are shown as photons/second/square centimeter/steradian. Mice treated with 19z1-CD80^**+**^ or 19-28z^**+**^ T cells showed relapsed disease at distinct anatomic sites including the periodontal region, CNS/calvarium and abdomen. (B) Bioluminescent tumor signal quantified per animal every week over a 120-day period. Quantification is the average photon count of the ventral and dorsal acquisition per animal at all given time points. One line represents one mouse. N = 7–8 mice per group or N = 15 for 19-28z^**+**^ T-cell group resulting from 2 pooled experiment (C) Survival is illustrated in the Kaplan-Meier curves. *P<0.05; **P<0.01; ***P<0.001. (D) Bone marrow hCD3^**+**^ T-cell absolute numbers evaluated 8 and 30 days after T-cell injection, as indicated. Each symbol indicates an individual mouse (n = 4). (E) Detailed bone marrow CD4^**+**^ and CD8^**+**^ T-cell absolute numbers gated on CD3^**+**^ cells showed in D.

To determine if the differences between 19-28z^+^ T cells and CD80-costimulated 19z1^+^ T cells observed *in vitro* would have an impact in a therapeutic setting, we compared their anti-tumor activity in a newly established systemic tumor model. NSG mice injected with 0.5x10^6^ GFP-FFLuc NALM/6 cells, a pre-B-cell ALL cell line negative for T costimulatory ligands, developed diffuse leukemia mainly homed in liver and bone marrow, as illustrated by bioluminescence imaging in Fig 2A and confirmed by FACS detection of CD19^+^ cells (data not shown). Without any treatment, mice developed hind limb paralysis at 18–20 days. Treatment with one administration of 2x10^5^ 19z1^+^ or 19z1-CD80^+^ T cells induced a significant but slight delay in tumor progression and eventually failed to eliminate the tumor (Fig 2A–2C). In contrast, the group treated with 19-28z^+^ T cells showed large tumor elimination and striking long-term survival improvement (Fig 2A–2C). Measures of human CD3^+^ cells counts in bone marrow 8 days after T cell injection showed that 19-28z+ T cells expanded 35-fold and 10-fold more than 19z1+ and 19z1-CD80+ T cells, respectively, and were still detectable one month after treatment (Fig 2D). Starting with a 1:1 ratio at the infusion time, both CD4 and CD8 subsets expanded similarly within one week in 19z1^+^ and 19-28z^+^ T cell groups whereas CD8^+^ T cell expansion was favored in the group treated with 19z1-CD80^+^ T cells (Fig 2E). Of note, we found more CD4 T cells than CD8 T cells in the 19-28z^+^ T-cell group, one month after treatment (Fig 2E). These results indicate that the 19-28z CAR triggers higher *in vivo* T-cell expansion than the 19z1 CAR, with or without CD80 costimulation, resulting in better tumor eradication.

### RNAi-mediated CTLA-4 down-regulation in CAR-targeted primary T cells

**Fig 3:**
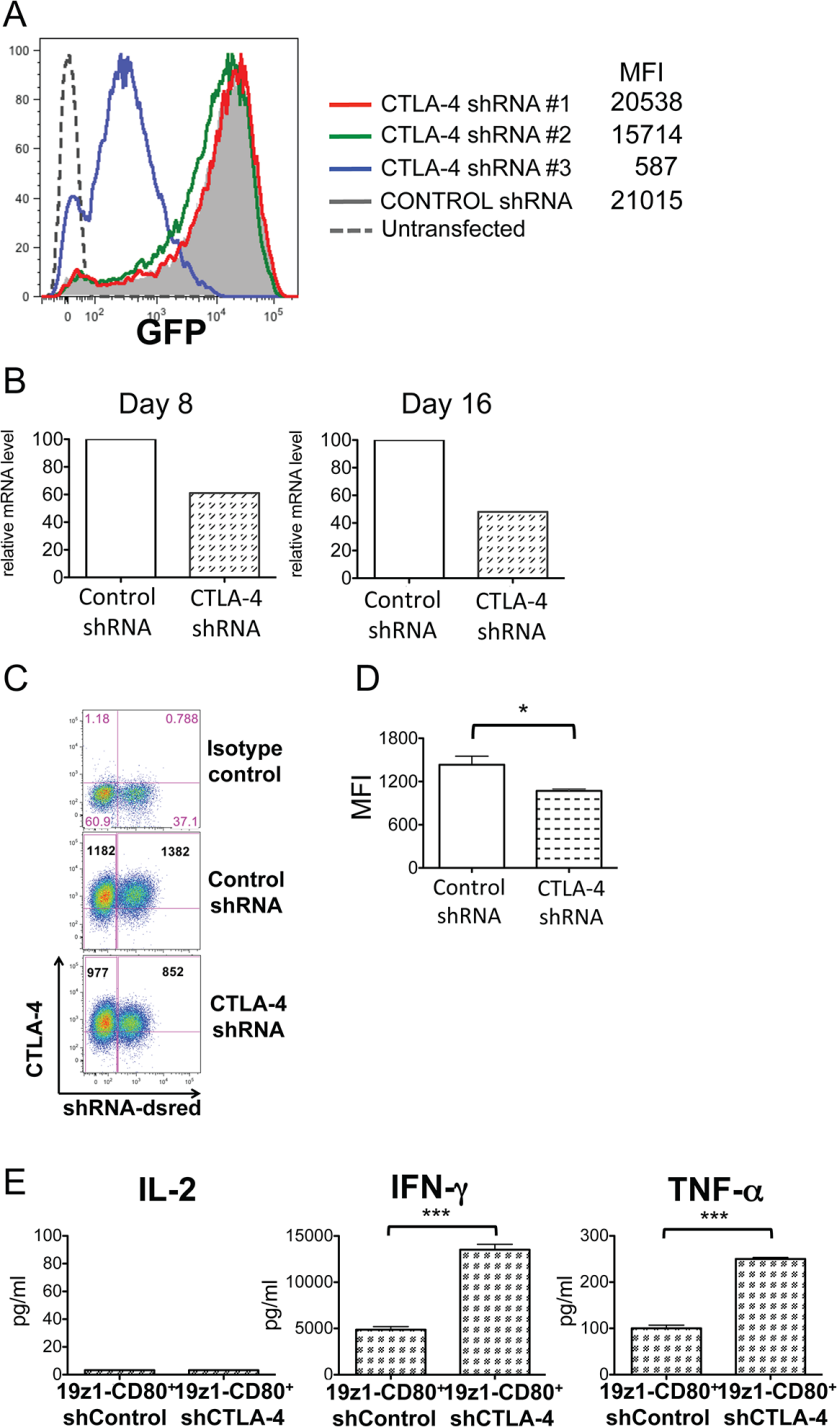
shRNA-mediated CTLA-4 down-regulation. (A) CTLA-4 knock–down transient assay. Briefly, 293 T cells were costransfected with CTLA-4/GFP and shRNAs/reporter plasmids at a 1:9 ratio. 48h later, MFI of GFP was evaluated by FACS analysis in the shRNA reporter gene gate. (B) Real-time PCR relative quantification of CTLA-4 transcript in 19z1-CD80^**+**^ T cells transduced to express a control shRNA or the anti-CTLA-4 shRNA #3 and *in vitro* stimulated with aAPCs expressing CD19, as illustrated in Fig 1. One representative out of 3 experiments is shown. (C) Flow cytometry analysis of intracellular CTLA-4 in 19-28z^**+**^ T cells transduced to express a control shRNA or the anti-CTLA-4 shRNA #3 and activated twice on aAPC. Plots from one representative donor are shown. Bold numbers indicate the MFI of the CTLA-4 staining in the shRNA negative (left) or positive (right) populations. (D) Histograms show the average of the measured CTLA-4 MFI gated in the dsRed-shRNA positive population in each T-cell group, from 3 different donors, *P* = 0.029. (E) Cytokines measured in supernatants 24h after coculture of 19z1-CD80^**+**^ T cells expressing the indicated shRNA on aAPCs expressing CD19. One representative out of 2 experiments is shown. *P* = 0.0002.

Since CD80 binds CTLA-4 after T cell activation, we hypothesized that the observed lack of persistence and poor anti-tumor efficiency of 19z1-CD80^+^ T cells could result from CTLA-4 inhibition. Each group of CAR-transduced T cells exhibited similar levels of total CTLA-4 protein before and after activation (data not shown). To investigate our hypothesis, we first evaluated the efficiency of three shRNAs targeting human CTLA-4 in an *in vitro* 293T cell based transfection system. As illustrated in Fig 3A, the shRNA #3 showed the highest level of CTLA-4 down-regulation and was subsequently used for studies in primary T cells. CAR-targeted T cells transduced with the vector encoding the CTLA-4 shRNA showed a 50% decrease in CTLA-4 mRNA levels (Fig 3B) and a 25% decrease in total protein level (Fig 3C). Although secretion of IL-2 was not affected, 19z1-CD80^+^ T cells expressing the CTLA-4 shRNA secreted more IFNγ and TNFα than the same T cells expressing a control shRNA upon *in vitro* antigen stimulation (Fig 3D).

### CTLA-4 down-regulation enhances anti-tumor ability and *in vivo* expansion of 19z1-CD80^+^ T cells, while not affecting anti-tumor activity of 19-28z^+^ T cells

**Fig 4:**
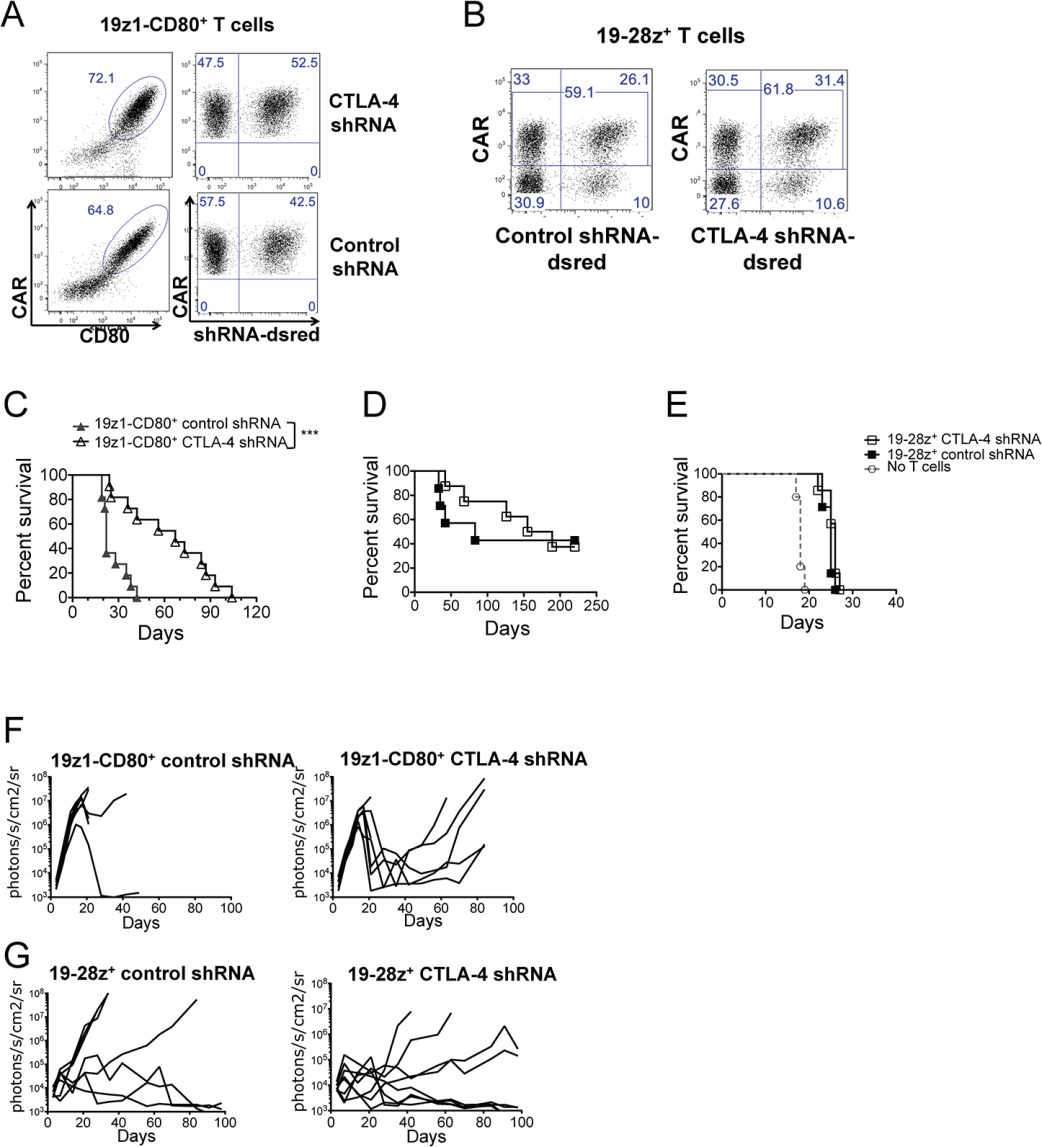
CTLA-4 down-regulation increases 19z1-CD80^+^ T-cell efficiency but not that of 19-28z^+^ T cells. (A-B) Facs plots showing expression of the CAR and shRNAs traced by dsRed in 19z1-CD80^**+**^ T cells (A) or in 19-28z^**+**^ T cells (B), generated from the same PBMC, before *in vivo* infusion. (C) Treatment of NALM/6 bearing NSG mice with 2x10^**5**^ 19z1-CD80^**+**^ T cells expressing an anti-CTLA-4 shRNA statistically enhanced survival when compared with similar treatment with 19z1-CD80^**+**^ T cells expressing a control shRNA, *P* = 0.0002. Kaplan-Meier curves include the results of two pooled experiments. N = 11 or 12 mice per group. (D-E) No statistical difference in survivals of NALM/6 bearing NSG mice treated with 19-28z^**+**^ T cells expressing an anti-CTLA-4 shRNA or a control shRNA at two different doses: 2x10^**5**^ (D) and 1x10^**5**^ T cells (E). (F-G) Tumor burden weekly quantified by bioluminescence imaging of NALM/6 bearing mice treated with 2x10^**5**^ 19z1-CD80^**+**^ T cells (F) or 19-28z^**+**^ T cells (G) expressing an anti-CTLA-4 shRNA or control shRNA. One line represents one mouse.

**Fig 5:**
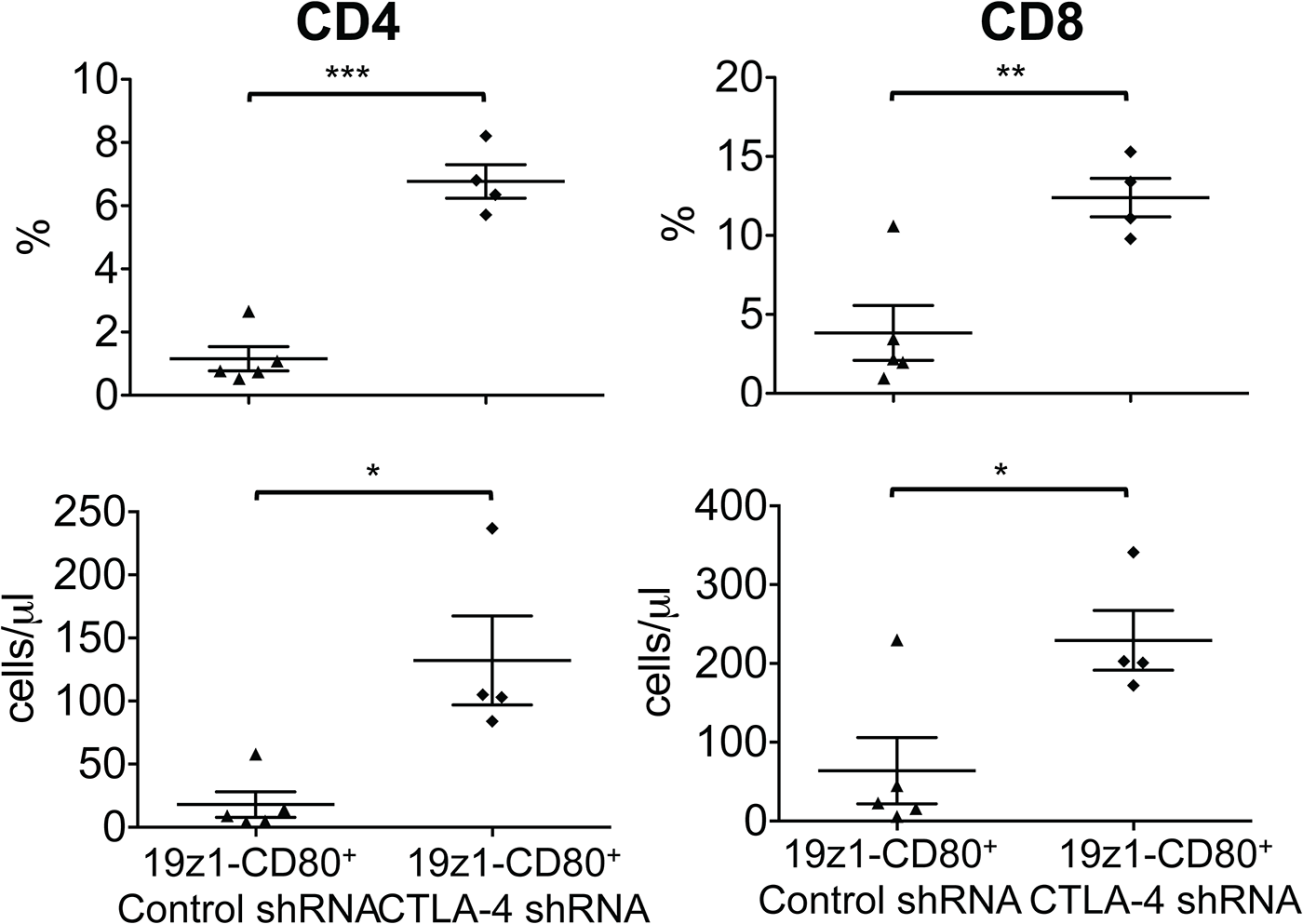
CTLA-4 down-regulation enhances *in vivo* expansion of 19z1-CD80^+^ T cells. Diagrams show percentages and absolute numbers of circulating human CAR^**+**^ CD3^**+**^CD4^**+**^ and CD3^**+**^CD8^**+**^ T cells as measured by flow cytometry 15 days after T cell injection in NALM/6 bearing NSG mice. Each symbol represents an individual mouse (n = 4). *P<0.05; **P<0.01; ***P<0.001.

Using the systemic tumor model described above, we assessed the anti-tumor capacity of 19z1-CD80^+^ T cells expressing a control shRNA or an anti-CTLA-4 shRNA, tracked by a fluorescent reporter (Fig 4A). 19z1-CD80^+^ T cells expressing a control shRNA failed to eliminate the tumor (Fig 4C and 4F), similarly to 19z1-CD80^+^ T cells (Fig 2). In contrast, mice treated with 19z1-CD80^+^ T cells expressing an anti-CTLA-4 shRNA had delayed tumor progression and highly improved survival, as shown in Fig 4C and 4F. Median survivals were 22 and 67 days for 19z1-CD80-Control shRNA^+^ and 19z1-CD80-CTLA-4 shRNA^+^ T cells, respectively (*p* = 0.0002). The therapeutic improvement was accompanied by increased numbers of circulating CD4^+^ and CD8^+^ 19z1-CD80^+^ T cells expressing the anti-CTLA-4 shRNA (Fig 5). Therefore, inhibiting CTLA-4 expression in 19z1-CD80^+^ T cells results in their enhanced anti-tumor efficiency.

To know if decreased CTLA-4 levels would affect anti-tumor properties of 19-28z^+^ T cells, we conducted similar experiments in which approximately 50% of the 19-28z^+^ T cells expressed the anti-CTLA-4 shRNA (Fig 4B), comparably to the shRNA transduced 19z1-CD80^+^ T cells (Fig 4A). Treatment of NALM/6 bearing mice with 2 or 1x10^5^ 19-28z^+^ T cells expressing the anti-CTLA-4 shRNA did not differ from treatment with the same dose of T cells expressing a control shRNA, as illustrated by comparable survivals and tumor burdens (Fig 4D, 4E and 4G). Since CTLA-4 down-regulation induces enhanced T-cell function in CD80 costimulated 19z1^+^ T cells, the absence of improvement in 19-28z^+^ T cells indicates that the latter show reduced sensitivity to CTLA-4-mediated T cell inhibition during their *in vivo* stimulation. Altogether, our results indicate that, upon CD19 recognition, 19-28z^+^ T cells are activated, expand and efficiently kill the targeted tumor cells while bypassing CTLA-4 inhibition.

## Discussion

In the present study, we compared for the first time *in vitro* and *in vivo*, the effect of antigen-dependent CD28 costimulation delivered through a second-generation CAR to CD80/CD28-mediated costimulation. Our studies confirm the superiority of 19-28z transduced T cells over 19z1 transduced T cells, previously reported in NALM/6 bearing SCID Beige mice [14,19] and in a clinical trial in lymphoma patients [12]. We also reconfirm that, as previously found with a second generation PSMA-specific CD28/CD3z-based CAR, the CD28-based CAR triggers less IL-2 secretion than a CD3-based CAR simultaneously stimulated with CD80 upon first exposure to antigen [13]. Significantly however, we find that, upon repeated exposure to antigen and adoptive transfer to tumor-bearing mice, 19-28z-CAR transduced human T cells display enhanced expansion and greater *in vivo* anti-leukemia efficiency than CD19-targeted T cells that are costimulated through CD28/CD80. In T-cell proliferation, cytokine secretion and *in vivo* tumor killing assays, we observed that CAR-mediated CD28 costimulation in 19-28z^+^ T cells is more potent than CD80 costimulation of 19z1 expressing T cells. To test the hypothesis that CTLA-4 accounts at least in part for this difference, we knocked down CTLA-4 expression specifically in retargeted primary T cells using shRNA. CTLA-4 knockdown in 19z1-CD80^+^ T cells increased secretion of IFNγ and TNFα but had no measurable effect on IL-2 levels. This may be due to the need for a higher threshold of activation to restore IL-2 secretion or immediate consumption of the produced IL-2 production. However, CTLA-4 knockdown enabled increased *in vivo* accumulation of 19z1-CD80^+^ T cells that might have been achieved through enhanced CD28 signaling independently of IL-2. The knock-down of CTLA-4, albeit partial, was evidently sufficient to trigger enhancement of 19z1-CD80^+^ T cells’ anti-tumor properties, although not modifying those of 19-28z^+^ T cells. These results emphasize the strength of CTLA-4 inhibition and its consequence on the anti-tumor abilities of CAR-targeted T cells. Indeed, by inhibiting CTLA-4 in 19z1-CD80^+^ T cells in which the CD28 signal comes from endogenous CD28 receptors activated by CD80 ligation, we almost reached the anti-tumor capacity of 19-28z^+^ T cells, in which CD28 costimulation is provided trough the CAR. Thus, the CD19-driven CD28 signal given in the absence of CD80 molecules is sufficient to trigger T-cell sustained proliferation and anti-tumor activity, which are unaffected by CTLA-4 inhibition. On the other hand, CD80-dependent CD28 activity is largely increased by CTLA-4 down-regulation. These results were expected since CTLA-4 inhibition occurs only in presence of its ligands, i.e. CD80 or CD86 [36]. Also, they corroborate those of two studies describing the cell-extrinsic inhibitory effect of CTLA-4. In murine models, adoptively transferred CTLA-4^-/-^ T cells displayed high *in vivo* expansion rates after antigen-specific stimulation, which were dampened by simultaneous co-transfer of wild-type antigen-specific CD4 T cells [37,38]. This CTLA-4-mediated ‘trans’ regulation may operate by limiting access to the B7 ligands through physical competition and/or trough the sequestration of the latter by CTLA-4 in intracellular vesicles, as elegantly demonstrated by Qureshi *et al* [27].

The cell-extrinsic regulation is emerging as the main mechanism of action of CTLA-4 [29]. It includes competition of the CTLA-4 extracellular domain with CD28 to bind CD80 or CD86 ligands, ligand transendocytosis [27] and triggering immunosuppressive effects through down-regulation of CD80 and CD86 in dendritic cells [29,39–41]. Thus, the physical interaction of the ectodomain of CTLA-4 with its ligands would be required for most of its inhibitory functions, with a cytoplasmic domain mainly involved in controlling quantity, cellular localization and recycling of the molecule [29,38,42].

While not improving the clinically used 19-28z CAR, our results demonstrate that CTLA-4 down-regulation can enhance the anti-tumor activity of adoptively transferred human T cells. These results extend those obtained in murine melanoma models, in which CTLA-4 blockade by an antagonist antibody of adoptively transferred anti-tumor CD4^+^ T cells enables large tumor eradication [43]. Interestingly, we observed increased *in vivo* peripheral T-cell counts in mice treated with CTLA-4-knockdown T cells. Increased absolute T-cell counts and melanoma-specific CTLs have also been measured in cancer patients receiving the anti-CTLA-4 mAb Ipilimumab and seemed to correlate with a clinical benefit [44–46]. Also, the remote administration of Ipilimumab in patients with melanoma treated by adoptive transfer of anti-tumor CD8^+^ T cells induced the expansion of the previously infused T cells [47]. The recently FDA approved drug has achieved therapeutic success inducing increased overall survival in patients with advanced metastatic melanoma in randomized phase III trials [45,48,49]. However, treatment with Ipilimumab is frequently accompanied with immune-related adverse events (IRAE) such as diarrhea, colitis, hepatitis, dermatitis and vitiligo that are severe in 10–25% of the patients at the highest dose and manageable with corticosteroids [45,48,50]. IRAEs are due to non-specific blockade of CTLA-4 in self-targeted T cells that destroy healthy tissues and are not dissociable from the beneficial anti-tumor effect. In fact, IRAEs often correlate with durable tumor responses [50]. Using a genetic approach that restricts CTLA-4 blockade to anti-tumor T cells, as we propose here, would avoid auto-immune side effects caused by a systemically injected mAb. Although not essential for CD28-based CAR transduced T cells, reducing CTLA-4 expression using shRNAs or other genetic technologies could be beneficial in TCR-transduced T cells currently used to target NY-ESO-1 or WT-1 [1,51]. One may envisage that such a decrease in intracellular CTLA-4 levels would decrease the T-cell activation threshold during contact with dendritic cells that express CD80 and CD86 and enhance their proliferation and anti-tumor abilities.

In summary, our study underscores that CD3z/CD28-based CARs are not subjected to CTLA-4 inhibition and demonstrates that genetic CTLA-4 down-regulation benefits human adoptive T cell therapy.
